# Designing Materials and Processes for Strong Polyacrylonitrile Precursor Fibers

**DOI:** 10.3390/polym13172863

**Published:** 2021-08-26

**Authors:** Hyunchul Ahn, Sang Young Yeo, Byoung-Sun Lee

**Affiliations:** 1 Advanced Textile R&D Department, Korea Institute of Industrial Technology, 143 Hanggaulro, Sangnok-gu, Ansan 15588, Gyeonggi, Korea; hahn@kitech.re.kr; 2 School of Polymer System/Department of Fiber Convergence Materials Engineering, College of Engineering, Dankook University, 152 Jukjeon-ro, Suji-gu, Yongin 16890, Gyeonggi, Korea

**Keywords:** polyacrylonitrile (PAN) fiber, mechanical properties, spinning process, microstructure, carbon fiber

## Abstract

Although polyacrylonitrile (PAN)-based carbon fibers have been successfully commercialized owing to their excellent material properties, their actual mechanical performance is still much lower than the theoretical values. Meanwhile, there is a growing demand for the use of superior carbon fibers. As such, many studies have been conducted to improve the mechanical performance of carbon fibers. Among the various approaches, designing a strong precursor fiber with a well-developed microstructure and morphology can constitute the most effective strategy to achieve superior performance. In this review, the efforts used to modulate materials, processing, and additives to deliver strong precursor fibers were thoroughly investigated. Our work demonstrates that the design of materials and processes is a fruitful pathway for the enhancement of the mechanical performance of carbon fibers.

## 1. Introduction

Carbon fibers have been considered the most promising reinforcement for composites used in various industries owing to their excellent material properties (e.g., high modulus (up to 900 GPa), high tensile strength (up to 7 GPa), and low density (1.75–2.00 g/cm^3^)) [1]. Their excellent mechanical properties encourage mechanical use, and thermal stability enables high-temperature applications. Remarkable electrical and thermal conductivities are also useful for expanding the applications of carbon fibers [2]. As such, carbon fibers have been extensively used in traditional applications, such as high-pressure durable containers for cutting-edge applications, including automobiles, aerospace, and high-end sports gears [3,4,5,6]. Global demand for carbon fibers is expected to reach 117 kt (kilotons) in 2022 [7], and the compound growth annual rate (CAGR) of the global carbon fiber market between 2017 and 2023 is 10.6% [8].

Commercially available carbon fibers are based on various precursors, such as polyacrylonitrile (PAN), pitch, and rayon [9], and their properties vary according to the precursor fibers [10]. PAN has been used as a precursor owing to its high melting temperature and high carbon yield in the range of 50–60% [11]. Pitch has emerged as a soft carbon precursor owing to its inherent aromatic molecular structure [12]. Rayon is another attractive precursor owing to its low cost [13]. Despite the high cost of the synthetic polymer precursor, PAN-based carbon fiber occupies the predominant carbon fiber market share of 90% based on its superior mechanical performance [14]. Commercially available carbon fibers from Toray and Hexcel are also mainly PAN-based products [15,16].

Although the pursuit of eco-friendliness and cost reduction by introducing biomass, such as lignin and cellulose, is an important research stream [17,18,19,20,21], advancing the PAN-based carbon fiber performance improvements and expanding their applications is another major research stream [22,23,24]. Despite the high carbon atomic content (> 92%) [25], the mechanical properties of PAN-based carbon fibers are still far below their theoretical values; specifically, they are less than 10% of the theoretical tensile strength of the carbon–carbon bond and less than 60% of the theoretical modulus of the graphitic microstructure [26]. This encourages research on advanced carbon fibers from PAN-based precursor fibers. It is well known that thermal treatments (i.e., stabilization, carbonization, and graphitization) are key processes used for the determination of the mechanical performance of carbon fibers: high carbonization (or graphitization) temperature is mainly for high modulus, while the low carbonization temperature with a long process time is for high strength [27]. However, pursuing both high modulus and strength cannot be achieved by the thermal treatment itself. Instead, it is necessary to design a strong precursor fiber to simultaneously achieve both performances. This review examines the recent studies on advanced precursor fibers for the synthesis of strong carbon fibers ranging from raw materials to the post-spinning process.

## 2. Overview of PAN Precursor Design Factors

**Figure 1:**
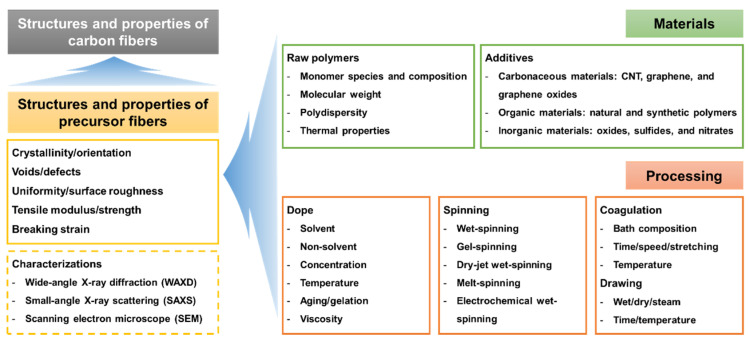
Overview of polyacrylonitrile (PAN) precursor fiber designing factors and characterization.

The design factors and representative characterizations of strong precursor synthesis are summarized in Figure 1. A strong carbon fiber design can be achieved via a good precursor fiber with a well-defined microstructure (e.g., high crystallinity, firm orientation, and low defect density) and morphologies (e.g., uniform diameter, circular cross-section, and low surface roughness). Crystallographic structures, such as crystallinity and orientation, were characterized by wide-angle X-ray diffraction (WAXD) [28], and voids or internal defect structures were examined with the use of small-angle X-ray scattering (SAXS) [29,30]. The surface and cross-sectional morphologies were investigated with the use of scanning electron microscopy (SEM). These microstructural and morphological features are complicated functions of materials and processing. From one perspective, well-designed materials can deliver excellent precursors and carbon fibers. It is clear that the polymeric structure of PAN, such as the copolymeric composition, molecular weight, and polydispersity, is the main parameter determining the precursor fiber properties. Their thermal properties are also important because carbon fiber manufacturing is always accompanied by thermal treatment. Employing adequate types and amounts of additives can be a key approach to the improvement of the mechanical properties of the precursor and carbon fibers. From another viewpoint, the processing from the dope preparation to the drawing process is another determinant of the mechanical performance of the precursor and carbon fibers. The cause–effect relationship between the factors and mechanical performance is discussed in detail in the following section.

## 3. Raw Polymers

### 3.1. PAN Structure and Molecular Weight

**Figure 2:**
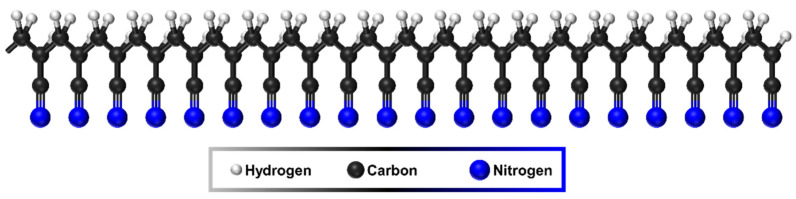
Schematic illustration of PAN molecular structure.

**Figure 3:**
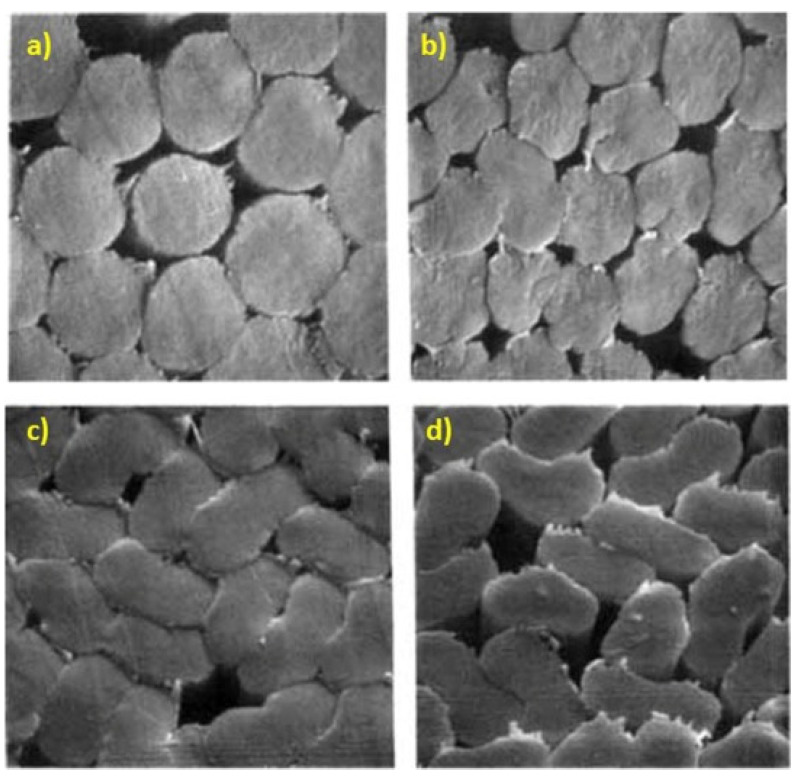
Effect of molecular weight on PAN cross-section, (**a**) 165,000, (**b**) 253,000, (**c**) 340,000, and (**d**) 429,000 g/mol (×1550) (reprinted with permission from [32]; copyright 1991 John Wiley and Sons).

The molecular structure of polymers is an important factor in determining the mechanical properties of the precursor and carbon fibers. PAN molecules consist of a hydrocarbon backbone and repeating nitrile chains, as shown in Figure 2. PAN molecular structures are simple but stereospecific, and these stereospecific polymers often have different thermal, mechanical, and chemical properties depending on their stereoregularity [31]. Conversely, the molecular structure is also a crucial factor in the rheological behaviors of the spinning dope. As such, attempts have been made to manipulate the molecular structures to obtain the optimum wet spinning process as well as the best mechanical properties of the precursor fibers. First, the simplest approach is to vary the molecular weights of the polymers. The molecular weight is directly related to the wet-spun precursor fiber morphologies owing to the effects on the viscosity, aging, and gelation behaviors of the spinning dope [32]. Figure 3 demonstrates the circular-to-cocoon-like cross-sections as the molecular weight increases. Despite the off-circular morphologies of the high-molecular-weight PAN fibers, the tensile strength of the precursor and carbon fibers increased with higher molecular weight owing to the increased crystal size and orientation. This was confirmed multiple times by other researchers [33,34].

Although the high-molecular-weight polymer has excellent mechanical properties, the high molecular weight generally induces increased viscosity and subsequent off-circular cross-section. A circular cross-section is preferable for preventing unwanted mechanical deterioration. To achieve this, reversible addition-fragmentation chain-transfer (RAFT) polymerization has been studied to increase the molecular weight without increasing the viscosity owing to the reduced polydispersity index (PDI) [35,36]. The improved mechanical properties of RAFT-polymerized, high-molecular-weight PAN fibers at a high concentration of dope were attributed to the improved rheological behaviors owing to the improvement of the molecular weight distribution [37,38]. Meanwhile, the ultra-high molecular weight inevitably causes high viscosity and poor spinnability. The low concentration and high extrusion pressure during the spinning process were employed to manufacture ultra-high-molecular-weight PAN fibers [39]. Despite the reduced dope concentration, the ultra-high-molecular-weight PAN precursor fibers exhibited extraordinary tensile properties, averaging 826 ± 129 MPa in strength and 16.5 ± 3.4 GPa in elastic modulus, with small filament diameters (5.3 ± 0.5 μm), while the diameter and tensile strength of the typical commercial PAN fibers were ≥6.3 μm and ≤690 MPa, respectively [40]. Thus, it could be concluded that a PAN polymer with a higher molecular weight is preferable to achieve better mechanical properties.

### 3.2. Copolymers

The introduction of comonomers to PAN polymers has been targeting two main goals: (i) acidic comonomers, such as acrylic acid, methacrylic acid (MA), and itaconic acid (IA), which were used to improve stabilization by reducing the cyclization temperature, and (ii) neutral comonomers, such as methyl acrylate (MA) and methyl methacrylate (MMA), and were employed to improve the solubility, drawability, and spinnability [41,42,43]. Comonomers, such as monobutyl itaconate (MBI) [44], vinylimidazole (VI) [45], and 2-acrtlamido-2-methylpropane acid (AMPS) [46], used in electrospun carbon fiber synthesis, are good candidates for improving the mechanical performance of carbon fibers. Superficially, introducing acidic comonomers improves spinnability by augmenting hydrophilicity, but a high content of acidic comonomer content results in reduced molecular weight during polymerization and poor spinnability [47]. In the meantime, the weakened intermolecular interactions caused by comonomer incorporation can be either beneficial or detrimental to the mechanical properties of the precursor fibers because of the improved spinnability and reduced crystallinity [41]. Such a copolymer also affects the mechanical properties, and the properties of the fiber vary depending on the copolymer composition under the same spinning conditions. For example, there was a study reporting a PAN-MA-IA terpolymer with doubled strength (16.87 cN/dtex) of the commercial-grade copolymer (PAN-MA (90 wt% AN and 10 wt% MA)) [48]. Thus, it is important to optimize the comonomer content to achieve strong carbon fiber properties by satisfying multiple aspects: good spinnability, high crystallinity, and optimum thermal transformable structure.

## 4. Spinning Process

### 4.1. Dopes

**Figure 4:**
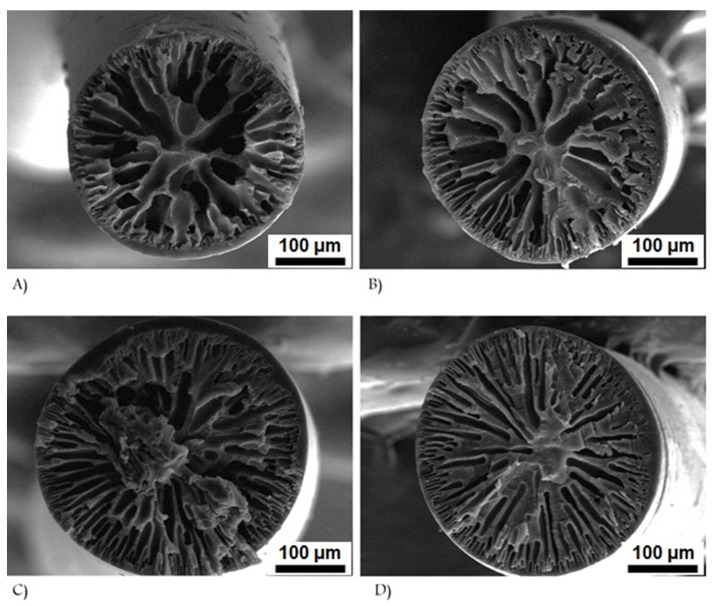
Effects of dope polymer concentration on cross-sectional morphology of as-spun PAN fibers: (**A**) 10 vol %, (**B**) 12 vol %, (**C**) 16 vol %, and (**D**) 20 vol % (reprinted with permission from [52]; copyright 2008 John Wiley and Sons).

The viscosity of the dope is one of the most important parameters for determining fiber morphology and properties. The molecular weight is the primary factor of viscosity [49]. The viscosity is directly related to the fiber morphologies and properties. The concentration and temperature are also important viscosity parameters [50,51]. Figure 4 shows that the number and length of the finger-like micrometer voids decreased as the polymer concentration increased [52], and fewer micrometer pores were preferable to achieve better mechanical properties. Note that a high concentration of PAN solution can cause aging effects, such as gelation, owing to entangled polymer molecules and intermolecular interactions [53].

**Figure 5:**
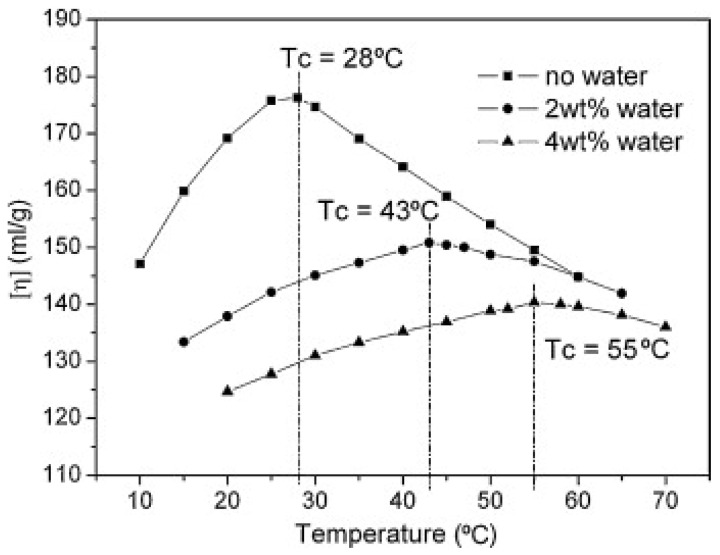
Temperature dependence of the intrinsic viscosity (η) for 23 wt% PAN/dimethyl sulfoxide (DMSO) solution at different water contents (reprinted with permission from [60], copyright 2009 Elsevier).

The solvent is another important component of the dope. Various studies have been conducted on the use of dimethyl sulfoxide (DMSO) because DMSO is the most extensively used solvent for manufacturing commercial carbon fibers [54,55]. Similar to DMSO, *N*,*N*-dimethyl formamide (DMF) and dimethylacetamide (DMAc) can dissolve PAN, but the rheological behaviors of the solutions with different solvents are not the same because of the intermolecular interaction between the PAN polymer and the solvent molecules [56]. By extension, the use of mixed solvents, such as DMSO and DMAc, was proposed as a potential strategy for the improvement of the mechanical properties that are based on the reduction of the viscosity and the improvement of spinnability [51]. Conversely, nonsolvents, such as water, have been used to delay the coagulation rate by reducing the rate of outflow and nonsolvent inflow in a rheological and thermodynamic manner [52,57,58]. The addition of a nonsolvent also causes rheological behavioral changes, including gel formation [57,58,59]. Figure 5 demonstrates the reduced intrinsic viscosity (η) and increased peak temperatures for PAN/DMSO solutions with higher water content [60]. Void size reduction and subsequent improvement in the mechanical properties were attributed to the optimum content of the nonsolvent. In fact, the addition of a nonsolvent to the dope is not the best option for achieving the highest mechanical performance for commercial use because there were significant voids and flaws associated with phase separation [61]. It should be mentioned that aqueous PAN solutions were prepared with the use of highly concentrated salts, such as NaSCN and ZnCl_2_, in water [62,63,64]. The use of water is desirable for protecting the environment and human beings, but the processability and performance need to be comparable to the as-is commercially major precursor fibers for replacing organic solvents.

### 4.2. Spinning Processes

Precursor fibers are manufactured commercially with the use of a wet spinning process or dry-jet wet spinning. The fibrous product is made by exchanging the solvents of the dope with a nonsolvent during the spinning process. The as-spun fibers are then rinsed several times, dried in air, stretched in steam, and wound at a certain tension. Fundamentally, inhomogeneous microstructural changes, such as the propagation of the crystallite orientation and crystallinity, increase from the skin to the core, and the volume fraction exchanged from the large pores to the small pores are accompanied by wet spinning and subsequent processes [65]. Thus, the processing parameters are closely related to the mechanical properties of the precursor fibers. For example, the extrusion rate results in shear-thinning-induced crystallization as well as surface-defect formation owing to insufficient coagulation [66]. Increasing the winding speed as a wet spinning process parameter affects the mechanical properties of the precursor and carbon fibers because of the insufficient microstructural development with low crystallinity [67].

**Figure 6:**
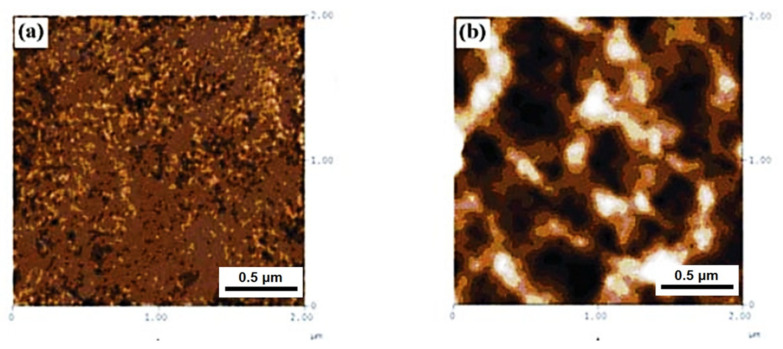
Atomic force microscope (AFM) micrographs of the spinning dopes: (**a**) raw and (**b**) gelled at 25 °C for 120 min (reprinted with permission from [69], copyright 2010 John Wiley and Sons).

**Figure 7:**
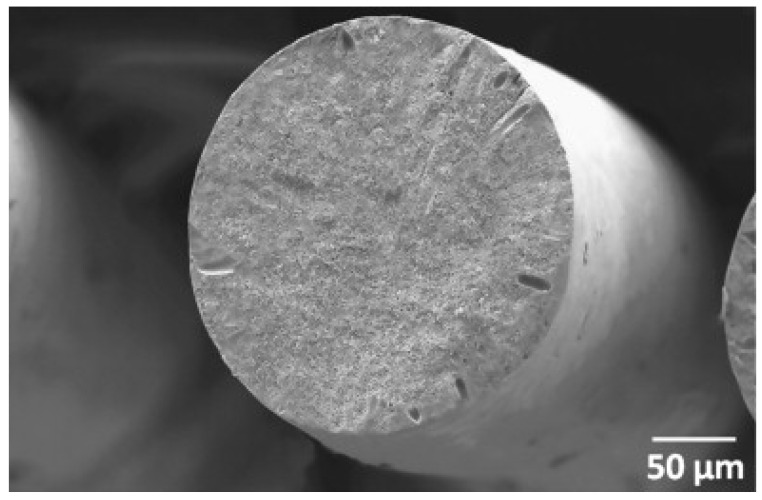
Scanning electron microscopy (SEM) images of the cross-section of the pregelled gel-spun fiber (reprinted with permission from [74], copyright 2011 Elsevier).

Gel spinning has been recently used to manufacture high-performance precursor fibers. The spinning set-up of gel spinning is fundamentally the same as wet spinning, but the difference mainly originates from the dope phase. Gelation of the dope is known to result from cross-linking in the presence of a nonsolvent [60,68]. Figure 6 shows the surface morphologies without (raw dope) and with (gelled dope) interconnected network structures [69]. Because the PAN dope gelation behavior depends not only on the composition but also on the thermal history of the solution and the experimental conditions [70,71,72], well-established experimental conditions from the dope preparation to the spinning process are crucial for controlling the quality and properties of the precursor fibers. The mechanical properties of the gel-spun fibers are expected to be excellent owing to their high orientation [73]. Indeed, the pregelled gel-spun fibers exhibited high crystallinity (e.g., 70.48% [74]) with a desirable cross-section (i.e., circular, low pore density, and little core/shell difference) (Figure 7) [71,75,76]. Gel-spun PAN fibers with a high molecular weight of 513,000 g/mol, representing a superb strength of 1.0 ± 0.1 GPa and a modulus of 20.7 ± 1.1 GPa with a breaking strain of 9.4 ± 1.5%, enabled remarkable tensile strength (5.5–5.8 GPa) and modulus (354–375 GPa) of the carbon fibers [26].

Although ordinary PAN molecules are spun into fibers via a wet spinning process because of the lack of melting behavior, previous attempts had been expended to enable the melt spinning process for manufacturing PAN fibers without using solvents, causing costs and environmental issues. The use of a melting point modifier, which consisted of water and acetonitrile, resulted in stable melt viscosities of the PAN copolymer, water, and acetonitrile mixture [77]. Melt-spun PAN copolymer fibers, which were prepared at a high copolymer content (e.g., methylacrylate, 15 mol%), exhibited fair mechanical properties, including a tensile strength of 260 ± 30 MPa, a Young’s modulus of 6.76 ± 1.78 GPa, and a breaking strain of 17.6 ± 0.84% [75]. Thus, there is still an adequate margin to improve the mechanical properties of the melt-spun precursor fibers to meet commercial standards.

**Figure 8:**
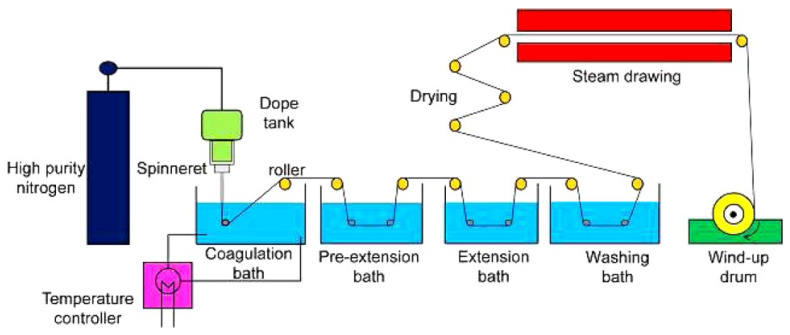
Schematic of the dry-jet wet spinning line (reprinted with permission from [76], copyright 2014 John Wiley and Sons).

Spinning setups were modified to improve the material properties of the precursor fibers. The dry-jet wet spinning process, schematically described in Figure 8 [76], is advantageous in the absence of influence of the spinnability by the coagulating conditions over the conventional wet spinning process owing to the existence of the air gap [78]. An air gap with a high jet-stretch ratio promotes molecular chain alignment and fiber diameter reduction [79]. Important factors that determine the mechanical properties of dry-jet wet-spun precursor fibers are the viscosity of the spinning dope, thermodynamic affinity, and draw ratio during the spinning process, and high viscosity and draw ratio with low thermodynamic affinity resulted in better mechanical properties [76]. Post-gelation (or aging) after the spinning process in cold methanol can be another step that can be used to improve the mechanical performance of dry-jet wet-spun precursor fibers [80].

**Figure 9:**
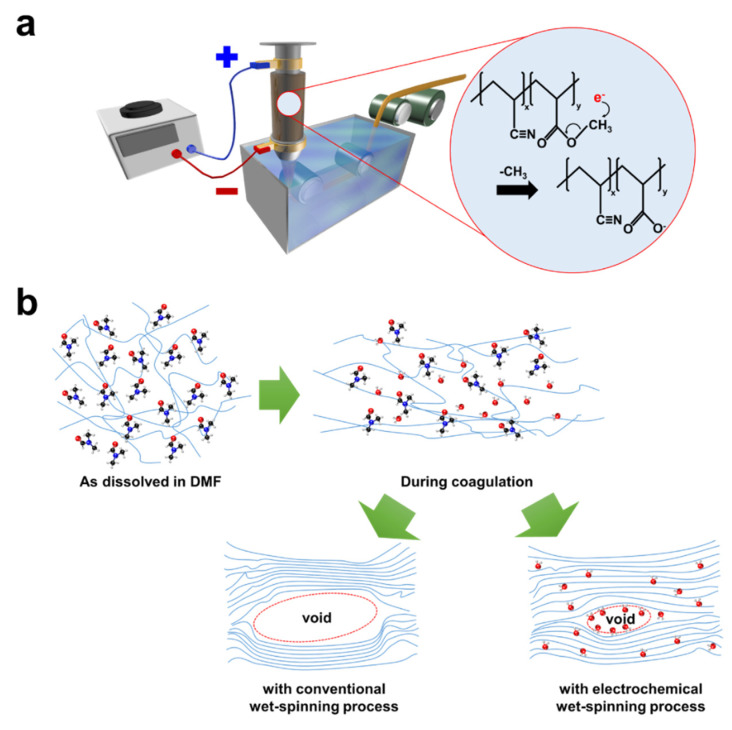
Schematics of (**a**) the electrochemical wet spinning process and (**b**) the molecular changes occurring therein. Note that no air gap was present between the spinneret and the coagulation bath (reprinted with permission from [81], copyright 2020 Elsevier).

A novel wet spinning process accompanied by an electrochemical reaction was recently reported in which electrochemical oxidation by the applied electric potential was designed to induce the plasticization effect of adsorbed water, as shown in Figure 9 [81]. Demethylation of the PAN copolymer increased the amount of water adsorption, and the adsorbed water helped reduce the voids and augment the orientation. As such, the tensile strength, modulus, and breaking strain simultaneously increased by 23.4, 23.5, and 28.1%, respectively, compared with the conventional wet-spun precursor fibers. It could be expected that additional work, such as coupling the electrochemical wet spinning with gel spinning, or dry-jet wet spinning, can synergistically improve the mechanical performance of the precursor fibers.

### 4.3. Coagulation

**Figure 10:**
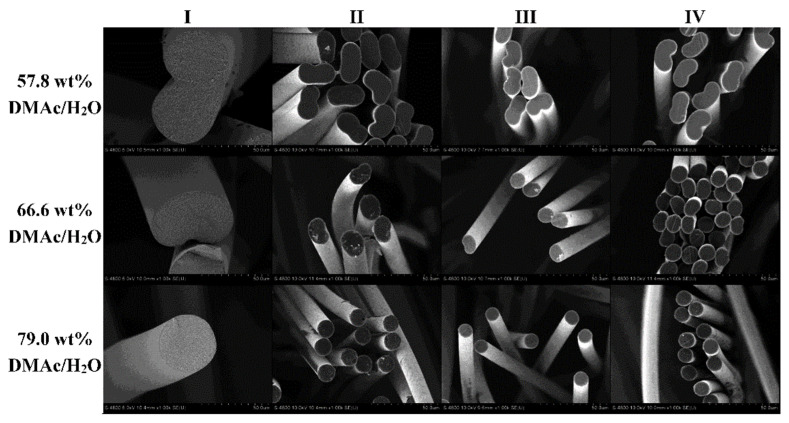
Effects of coagulation bath composition at approximately 19 °C on fiber shape and size as the fiber progresses down the spinning line [90].

Coagulation is another crucial process because the morphologies of the precursor fibers are fixed to the protofibers [82]. Coagulation is fundamentally attributed to the exchange of the outflow solvent and the influent nonsolvent, and the inhomogeneity of the solvent concentration distribution results in dense skin and loose cores [83,84,85]. The key parameters of the coagulation step are the composition [86,87], temperature [88], and time [89] of the coagulation bath. A higher concentration of the solvent in the low-temperature coagulation bath at around 19 °C was preferable because of the improved morphologies, such as the increased circular cross-section and reduced diameter (Figure 10), improved molecular orientation, and advanced mechanical performances [90], while there was an optimum solvent concentration of around 70% where the coagulation temperature was maintained at 50 °C. The crystallinity was maximized, while the defects of the cross-section and surface were the least at the optimum concentration of 70% [86]. Sufficient coagulation time is required because a short coagulation time results in a loosely packed protofiber microstructure [91]. The dry-jet wet-spun precursor fiber demonstrated a smooth surface owing to the macromolecular recovery before coagulation, while the conventional wet-spun fibers showed a fibril-structured surface [92]. It is noteworthy that coagulation is a useful step for the introduction of chemical modifiers, such as ammonium iron sulfate, for better thermal treatment [93].

### 4.4. Post-Spinning Process: Drawing and Densifying

**Figure 11:**
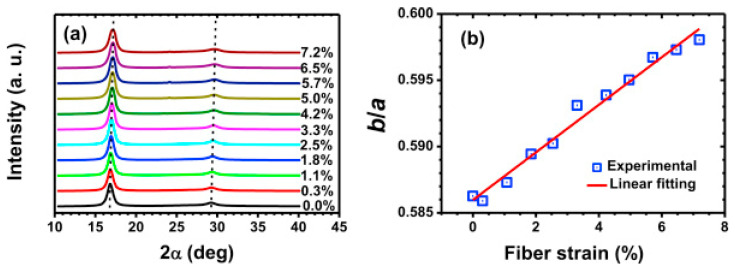
(**a**) Equatorial scans of PANFs at different fiber strains. Dashed lines guide the change in peak positions. (**b**) Variation of b/a value as a function of the fiber strain of PANFs. The listed symbols denote the experimental value, and the solid line is a linear fitting to the experimental values from calculated in situ 2D wide-angle X-ray scattering (WAXS) results (reprinted with permission from [96], copyright 2014 Elsevier).

The post-spinning process is mainly designed to densify and align the microstructure with superb mechanical properties. Although the microstructure of PAN precursor fibers is mostly arranged during the spinning process, the crystalline structural rearrangement to more ordered crystals followed by increased tensile strength and modulus are attributed to the densification treatment [94]. In addition, additional drawing (or stretching) processes enable high strength, high breaking strain, and high toughness owing to the improved molecular orientation [95]. In situ microstructural changes subject to drawing conditions showed that the orthorhombic unit cell of the PAN crystalline structures anisotropically transformed to a tetragonal unit cell with a reduced interdistance between the PAN molecular chains as the strain increased: (110) and (202) peaks at approximately 2θ = 17° and 29° shifted to higher angles (Figure 11a), and the lattice parameter ratio (b/a) increased linearly (Figure 11b) [96]. The peak positions shifted to slightly higher angles as the PANF tensile strain increased, indicating a compact arrangement of PAN molecular chains. Moreover, the change in the lattice parameter ratio reveals the microstructural change in the orthorhombic unit cells to the tetragonal unit cells as the macroscopic tensile strain of PANF increases. The material properties of drawn fibers are highly dependent on the processing temperature and drawing ratio; high temperature and high drawing ratio are preferable owing to the enhanced chain mobility and improved crystalline orientation [97,98,99]. The drawing effects on the microstructural changes and mechanical property improvements have been demonstrated with the dry-jet wet spinning process [100,101]. To determine the most effective drawing process, the microstructures and mechanical properties of the wet- and dry-drawn precursor fibers were evaluated, and the wet-drawn precursor fiber exhibited better modulus and rigidity, while the dry-drawn precursor fiber exhibited slightly higher strength and breaking strain [102]. Therefore, the combination of wet and dry drawing processes can help optimize the microstructure and mechanical performance. Novel approaches, such as hot stretching in a supercritical carbon dioxide (CO_2_) atmosphere, have also been attempted to improve the crystallinity as well as the mechanical properties, but added efforts ought to be expended to employ this concept in the continuous process [103]. Based on these efforts, the post-spinning process has been set in three stages: (i) wet-fiber stretching (working below the glass transition temperature (T_g_) in water or water/DMSO mixed solution medium), (ii) high-temperature densification (working above T_g_), and (iii) steam stretching (working at around T_g_ in high-temperature and high-pressure vapor) [104].

## 5. Functional Additives

**Figure 12:**
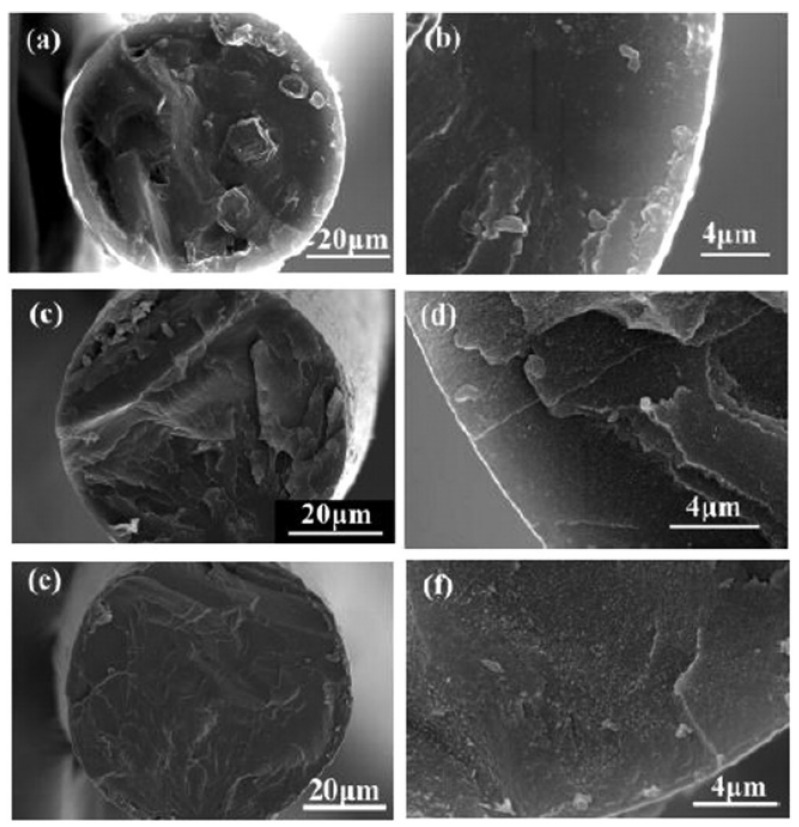
SEM micrographs of various as-spun PAN fibers containing different MWCNT contents ((**a**,**b**): 0; (**c**,**d**): 0.5 wt%; (**e**,**f**): 1.0 wt%) (reprinted with permission from [110], copyright 2011 John Wiley and Sons).

The mechanical performance of the precursor and carbon fibers can be further improved by compositing with additives. Compositing with graphitic nanocarbons, such as carbon nanotubes (CNTs) and graphene, is the most easily accessible route used to improve the precursor and carbon fibers owing to their exceptional mechanical performance (e.g., Young’s modulus of ca. 1 TPa and tensile strength of 0.15 TPa from the *sp*^2^ hybridized carbon basal plane) [105]. The addition of CNTs results in a change in the rheological behavior as well as an enhancement in the mechanical properties of the precursor fibers [106,107]. Single-wall carbon nanotube (SWNT) 10 wt% composite precursor fiber exhibited a double Young’s modulus (16.2 ± 0.8 GPa) and 43% increased tensile strength (0.33 ± 0.02 GPa) in comparison with those of the raw precursor fibers [108]. Young’s modulus and tensile strength of gel-spun PAN fibers increased to 19.2 ± 2.9 GPa and 1.01 ± 0.07 GPa, respectively, following the addition of four-walled carbon nanotubes (FWNTs) 1 wt%, while those of the pristine PAN fibers were 16.6 ± 1.6 GPa and 0.80 ± 0.11 GPa [109]. The dispersion of CNTs in the polymer matrix was an important issue in the 2000s. A careful study was conducted to determine the dispersion as follows: (i) multiwalled carbon nanotubes (MWNTs) were chemically treated in a concentrated HNO_3_/H_2_SO_4_ mixture, (ii) NaOH was slowly added to the mixture to adjust the pH of the mixture to neutral, (iii) the mixture was purified by repeated centrifugal sedimentation and ultrasonic dispersion in deionized water and dried at 40 °C in the presence of a vacuum, (iv) the chemically treated MWNT was dispersed in water at a concentration of 2 mg/mL, (v) PAN polymer was carefully added to the MWNT-dispersed water, and vi) the mixture was shear homogenized at 20,000 revolutions per minute with the use of a high-shear dispersing emulsifier and dried at 40 °C before the dope preparation [110]. The chemically treated MWNT 0.5 wt% and 1 wt% contained in the dry-jet wet-spun precursor fibers did not affect the circular and dense cross-sectional morphologies and homogeneity (Figure 12). Instead, the MWNTs contributed to the crystallinity and crystallite size increase following the improvement in tensile modulus and strength (i.e., 11.4 and 0.906 GPa) in comparison with those of the raw precursor fiber (i.e., 7.02 and 0.761 GPa).

Graphene (an emerging carbon nanomaterial) has also been composited to the precursor fibers. Graphene oxide-driven graphene via chemical reduction with hydrazine and ammonia was added to the dope with a concentration of 0.5 wt% in solid content [111]. The use of *N*-isopropylacrylamide (NIPAM) comonomer and a not fully optimized process may cause the unsatisfactory mechanical performances of the raw precursor fibers (E = 60.3 MPa and σ = 2.9 MPa), but the graphene addition significantly helped improve the mechanical performances (E = 68.7 MPa and σ = 3.6 MPa) compared with those of the raw fibers. Recently, PAN-based precursor fibers that contained small amounts of graphene prepared through a modified shear exfoliation method exhibited gradual improvement in the mechanical properties: the modulus increased from 3.5 to 6 GPa, and the strength increased from 40 to 80 MPa as the graphene content increased from 0 to 0.1 wt% [112]. Surprisingly, the addition of 0.075 wt% graphene to the PAN resulted in the 225% increase in strength and 184% enhancement in Young’s modulus compared with the raw PAN-based carbon fibers. Meanwhile, graphene oxides were employed as the mechanical reinforcing additive [113]. The optimum Young’s modulus (11.24 GPa) and strength (118 MPa) were exhibited at a graphene oxide concentration of 1 wt%. Even though the graphene (or graphene oxide) addition effects to the precursor fiber mechanical performance were considerable, the reported mechanical performances are still not close to the well-established PAN precursor fibers without additives (discussed in Section 3 above).

**Figure 13:**
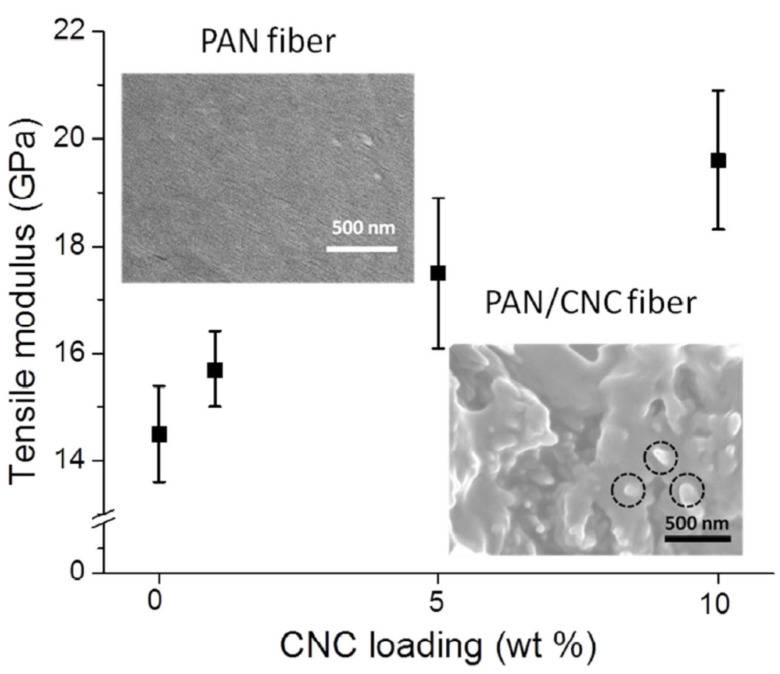
Young’s modulus change as the cellulose nanocrystal (CNC) content increased. Insets are SEM images of fracture surface of fibers of PAN and PAN/CNC 10 wt% (reprinted with permission from [114], copyright 2015 American Chemical Society).

Organic additives can also be used as mechanical reinforcements. Cellulose nanomaterials with high tensile strength (7.5 GPa) and modulus (110–220 GPa) have been applied as low-cost and biobased alternative reinforcements [114,115]. By compositing cellulose nanocrystal to the PAN matrix up to 10 wt%, Young’s modulus of the precursor fibers was increased from 14.5 to 19.6 GPa according to the rule of mixture (Figure 13), and the tensile strength improved from 624 to 709 MPa owing to the microstructural changes, such as better chain alignment and crystallinity increases from 50 to 62% [114]. It was confirmed that the cellulose nanocrystal reinforcements also contributed to the advancement of the mechanical performance of carbon fibers [115,116]. In fact, the main purpose of the search for biomass additives, such as cellulose, lignin, and alpaca fiber, is eco-friendliness and cost effectiveness [114,117,118,119,120,121,122,123,124]. It is inevitable to avoid deterioration of the mechanical performance following the composition with the biomass-derived additives owing to the low carbon atomic content and ineffective molecular structure that is intended to be carbonized. As such, little deterioration in mechanical performance is required. A high-loading cellulose nanocrystal composite (~40 wt%) precursor was designed, and its mechanical performance was investigated [125]. The comparable mechanical performance of the high-loading composite precursor to the raw PAN fiber provides a potential for manufacturing biomass-derived carbon fibers.

Other organic substances have also been used as additives. Acrylamide monomer was blended in the PAN dope to promote oxidative stabilization. This compound also caused the increase in the Young’s modulus of the precursor fiber from 3.03 to 5.54 GPa at an acrylamide concentration of 5 wt% [126]. The increase resulted from the better molecular orientation owing to the plasticizing effect of the acrylamide, and the mechanical property enhancement of the precursor fiber was directly translated to the mechanical performance of the carbon fibers. Blending with polyimide (PI) resulted in improved mechanical performance, but PAN was used as an additive rather than a matrix [127,128]. Therefore, it is necessary to assess the effects of blending PI as an additive.

Inorganic nanomaterials were also examined for the mechanical reinforcement of PAN precursor fibers. The addition of 1 wt% silica resulted in a Young’s modulus of 5.94 GPa and a tensile strength of 1.07 MPa, while the raw precursor fiber exhibited a Young’s modulus of 2.82 GPa and a tensile strength of 0.286 MPa [129]. The flower-like MoS_2_-SiO_2_ nanohybrids/PAN precursor composite showed a 42% increase in tensile strength (55.9 cN) based on the significantly increased crystallinity (58.42%) as well as the improved flame retardant performance [130]. Similarly, the nitrogen-phosphorous-zinc-containing sandwich-like MoS_2_ hybrids/PAN precursor composites exhibited a 68% increase in tensile strength (84.6 cN) with increased crystallinity (67.87%) and improved flame retardant performance [131]. The addition of AgNO_3_ and ascorbic acid to form the Ag nanoparticles (25 nm) resulted in a crystallinity change (from 40.9 to 56.4%) and increase in the mechanical performances (the modulus increased from 583.49 to 850.81 cN/tex, and the strength increased from 38.92 to 41.17 cN/tex) [132]. The addition of 1 wt% TiO_2_ and 3 wt% AgNO_3_ led to improved mechanical performance as well as multifunctionality: tensile strength (8.72 cN/tex), conductivity (10^−4^ S/cm), antibacterial activity, and photocatalytic activity [133]. Thus, the addition of inorganic nanomaterials contributes not only to the improvement of mechanical performance but also to novel functionality.

The mechanical properties of the PAN precursor fibers are listed in Table 1. From the thorough investigation of the mechanical performances of the existing research, it can be concluded that high molecular weight and slow coagulation with an adequate amount of additive resulted in a superior modulus as well as strength owing to the high crystallinity with fewer defects. It should be mentioned that numerous attempts have been expended to improve the mechanical properties by introducing ceramic additives, and the effects were significant (generally double-digit improvement was represented). However, the absolute values were far below those of the precursor fibers from well-established spinning processes. Thus, the effect needs to be re-examined subject to the existing high-strength PAN fiber spinning processes.

## 6. Conclusions

The current review summarized recent efforts that had been expended to design PAN precursor fibers for mechanically advanced carbon fibers. Despite more than three decades of studies and commercialization legacies, the mechanical properties of PAN-based carbon fibers are still considerably lower than those of the theoretical values. Thus, numerous attempts have been conducted to improve the microstructural and morphological perfection of the precursor and carbon fibers that have been based on the increase in the molecular weight, the introduction of a new copolymer, optimization of the doping composition, the design of a new spinning process, exploration of the best drawing process, and compositing additives. The high molecular weight of the PAN and gel spinning process resulted in extraordinary mechanical performances owing to the considerable improvement in the microstructures. Other attempts, such as the modified wet spinning process and additive addition, also contributed to the mechanical performance improvement, but the absolute strength and modulus were less significant than those from the molecular weight and gel spinning process. Thus, it is necessary to demonstrate the approaches using the well-established material and process for validating the actual feasibility. In fact, it is expected that the theoretical strength and modulus of the carbon fiber can be achieved if the perfect crystal with the uniaxially aligned PAN molecules along the precursor fiber axis is designed. In this regard, the enhancing electrostatic attraction between the more electronegative nitrile group and the less electronegative hydrocarbon backbone can be a key approach to develop a more compact microstructure with increased orientation enabling closer values of the carbon fiber mechanical properties to the theoretical values.

## Figures and Tables

**Table 1:** Representative mechanical properties of PAN precursor fiber (DMAc: dimethylacetamide; DMF: *N*,*N*-dimethyl formamide; DMSO: dimethyl sulfoxide; SWNT: single-wall carbon nanotube; MWNT: multiwalled carbon nanotubes).

Researchers	Modulus (GPa)	Strength (MPa)	Dope Concentration (wt%) and Solvent	Spinning Process	CF Modulus (GPa)	CF Strength (MPa)	Notes
Morris et al. [40]	16.5 ± 3.4	826 ± 129	6.5%, DMAc	Dry-jet gel	345	4300	Ultra-high-molecular-weight PAN
Chae et al. [26]	20.7 ± 1.1	1000 ± 100	~10.5%, DMF	Dry-jet gel	375	5800	Low-temp coagulation get spinning
Alcalá-Sánchez et al. [48]	-	16.87 cN/dtex	20%, DMF	Wet	-	-	Terpolymerization
Lee et al. [76]	6.76 ± 1.78	260 ± 30	-	Melt	110	1370	Melt spinning
Min et al. [108]	16.2 ± 0.8	330 ± 20	~13.8%, DMAc	Dry-jet wet	-	-	SWNT
Liu et al. [109]	19.2 ± 2.9	1010 ± 70	~15%, DMF	Dry-jet gel	-	-	MWNT
Gao et al. [112]	6.0	80	~7.5%, DMSO	Wet	233	1919	Graphene
Zhao et al. [113]	11.24	118 ± 2	15%, DMF	Wet	-	-	Graphene Oxide
Chang et al. [114]	19.6 ± 2.3	709 ± 98	~ 13.5%, DMF	Dry-jet wet	-	-	Cellulose nanocrystals
Yusof et al. [126]	5.54 ± 0.03	-	~18%, DMF	Dry-jet wet	35	-	Acrylamide
Mataram et al. [129]	5.94	1.07	DMF	Dry-jet wet	-	-	Silica
Peng et al. [131]	-	19.16 ± 0.45	20%, DMF	Wet	-	-	Molybdenum disulfide
Karbownik et al. [132]	850.81 cN/tex	41.47 cN/tex	23%, DMF	Wet	-	-	Silver nitrate

## Data Availability

Not applicable.
